# Schizophrenia and Increased Distrust-Based Competitiveness in Interpersonal Interactions: A Serial Process Model

**DOI:** 10.1093/schbul/sbad021

**Published:** 2023-03-13

**Authors:** Lyn Ellett, Tim Wildschut, Paul Chadwick

**Affiliations:** School of Psychology, University of Southampton, Southampton, UK; School of Psychology, University of Southampton, Southampton, UK; Department of Psychology, University of Bath, Bath, UK

**Keywords:** paranoia, schizophrenia, prisoner’s dilemma game, competition, persecutory delusions, distrust

## Abstract

**Background and Hypothesis:**

Game theory paradigms, such as the Prisoner’s Dilemma Game (PDG), have been used to study nonclinical paranoia, though research using clinical populations has been scarce. We test our novel theoretical model that schizophrenia leads to competitiveness in interpersonal interactions, and that this link is serially mediated by trait paranoia, state paranoia, and distrust.

**Study Design:**

In this quasi-experimental study, individuals with schizophrenia spectrum diagnoses with current persecutory delusions (*n* = 46) and a nonclinical control group (*n* = 43) played the PDG, and completed measures of trait paranoia, state paranoia, and distrust.

**Study Results:**

Individuals with schizophrenia competed more in the PDG than the control group. Supporting our theoretical model, all direct effects were significant: schizophrenia was associated with higher trait paranoia (H1); trait paranoia predicted state paranoia in the PDG (H2); state paranoia in the PDG predicted distrust of the opponent in the PDG (H3); and distrust predicted competition in the PDG (H4). The hypothesized indirect effect of schizophrenia on competition in the PDG via trait paranoia, state paranoia, and distrust was supported in a serial mediation model (H5).

**Conclusions:**

The findings make clear theoretical and methodological contributions. We provide the first evidence for a theoretical process model by which schizophrenia leads to competitiveness in interpersonal interactions via trait paranoia, state paranoia, and distrust. Game theory paradigms, and the PDG in particular, are important for advancing theory and research on paranoia as it occurs in both clinical and nonclinical populations.

## Introduction

Persecutory delusions are beliefs that other people are intentionally trying to cause one harm^1^ and are part of the symptom profile of schizophrenia. Milder forms of persecutory thinking, often referred to as “paranoid beliefs” or “nonclinical paranoia,” have been shown to be common in the general population.^2–5^ The data on nonclinical paranoia are consistent with prevailing theoretical models proposing continuity between nonclinical and clinical experiences^6,7^ and with an evolutionary perspective on paranoia, which proposes that the capacity to perceive others as malevolent may have been selected because it held survival value in ancestral environments.^2^ Experimental research has sought to elucidate the key mechanisms involved in the etiology and maintenance of persecutory thinking, as it occurs in both clinical and nonclinical populations. Whereas a range of paradigms have been developed and tested with nonclinical groups, experimental research with clinical populations remains scarce.

Game theory paradigms are increasingly being used to study (nonclinical) paranoia, with research to date utilizing the Prisoner’s Dilemma Game (PDG),^8,9^ the Dictator Game (DG),^10–12^ and the Trust Game (TG).^13^ In both the DG and the TG, one player first decides how to split an endowment between themselves and the other player. In the DG, the recipient then decides whether to accept or reject the offer (rejection results in both players receiving a payoff of 0). In the TG, the initial proposed endowment is increased before it is received (usually doubled or trebled) and the recipient must choose whether to keep the full amount without repercussion, or whether to send some of it back to the other player. To date, game theory paradigms have been used to understand how individual differences in nonclinical paranoia relate to behavioral outcomes through associations with trait and state paranoia,^8,9^ including measuring harmful attributions towards the other player in the game.^13^

In the PDG, individuals make a forced choice to either cooperate or compete with the other player, with payoffs dependent on the choice made by both players. On each trial, if only one player competes, that competitive person receives the maximum possible reward and the cooperative other player gets the minimum possible reward; if both compete their rewards are the same but lower than if both cooperated. In the PDG, decision-making is thus strongly influenced by perception of the other’s likely behavior towards the self, making it an ideal paradigm for studying paranoia. Trust is crucial for establishing mutual co-operation, and distrust (the expectation that the other player will choose to compete in the game) promotes competition. Research has shown that competition in the PDG is indeed associated with state paranoia.^9^ However, in the PDG, competition might reflect either greed (i.e., predicting that an opponent will cooperate, and responding exploitatively to this possibility) or distrust (i.e., predicting that an opponent will compete, and responding defensively to this possibility^14^). Theoretical models of paranoia would predict an association between paranoia and distrust-based—but not greed-based—competition. PDG research has shown that state paranoia is associated specifically with distrust-based competition, and not with greed-based competition.^9^ Distrust-based competition in the PDG has therefore been proposed as a behavioral marker or signature of nonclinical paranoia.^9^

Game theory paradigms are thus providing a unique window into paranoia-relevant thinking and behaviors. A strength of these paradigms is that they are interpersonal (paranoia by definition is an interpersonal phenomenon as it involves perceived threat to self from others) and involve genuine social interactions with others^15^ who are outside of the individual’s persecutory delusion system. Game theory paradigms have the further advantage of modeling some of the necessary environmental conditions that give rise to persecutory thinking, such as ambiguity^16^—for example, in the PDG, a player is unaware of their opponent’s choice at the time of making their own choice.^9^ This latter point is a crucial strength of the PDG paradigm when studying paranoia and may shed light on why paradigms such as the Dictator Game (DG), in which one of the players moves first, have not also found an association between paranoia and distrust-based competition.^17^ Inconsistent findings concerning how paranoia relates to distrust and competition may also reflect important differences in the measurement of paranoia across studies. Studies using the DG and UG have measured trait paranoia at baseline (sometimes several months prior to playing the experimental game) but not measured state paranoia specifically about the opponent either during or after the game.^17^ Studies using the PDG have measured both baseline trait paranoia and state paranoia about the opponent.^8,9^ Including both measures might arguably provide a more fine-grained assessment of paranoia in game contexts.

## The Current Study

**Fig. 1.**
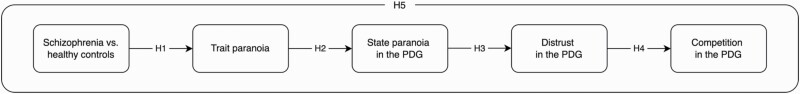
The Process Model Linking Schizophrenia to Competition in the PDG: Hypothesized Direct Effects (H1-H4) and Serial Indirect Effect (H5).

To date, no study has applied a game theory paradigm to study paranoia in a clinical population. In the current study, individuals with current persecutory delusions within a schizophrenia spectrum diagnosis are compared with controls on the PDG. We are particularly interested in understanding how persecutory delusions within the context of schizophrenia spectrum disorders may affect people in their social interactions, focusing specifically on how they might influence decision-making (behavioral choice in the PDG). We therefore expect individuals with persecutory delusions to choose to compete more in the PDG (rather than cooperate) than nonclinical controls, because competition can arise from the perception that the other player possesses malevolent intentions, which is a key defining feature of persecutory delusions. Figure 1 summarizes our proposed theoretical model. Based on the theory and evidence reviewed above, we predict that: schizophrenia will be associated with higher trait paranoia (H1); trait paranoia will predict state paranoia in the specific context of the PDG (H2); state paranoia in the PDG will predict distrust of the opponent in the PDG (H3); and distrust in the PDG will predict the behavioral choice of competition (H4). Finally, H1-H4 imply an indirect effect of schizophrenia on competition in the PDG via trait paranoia, state paranoia, and distrust (H5). H5 is the key prediction of our serial process model.

## Method

### Participants

We recruited 2 groups of participants—people with current persecutory delusions and a schizophrenia spectrum diagnosis (*n* = 46) and nonclinical controls (*n* = 43). Inclusion criteria for the clinical group were: (1) a diagnosis of a Schizophrenia-spectrum disorder and experiencing current persecutory delusions (confirmed by a consultant psychiatrist); (2) aged over 18 years of age. Exclusion criteria were: (1) organic cause for symptoms; (2) diagnosis of a learning disability; (3) absence of distressing persecutory delusion. Diagnoses were Paranoid Schizophrenia (*n* = 9), Schizoaffective Disorder (*n* = 7), and Schizophrenia (*n* = 30). We recruited clinical participants from secondary care mental-health services in the National Health Service in England. In the nonclinical control group, inclusion criteria were: (1) aged 18 or over; (2) no current or previous mental health diagnosis; (3) no history of contact with mental health services. We excluded one female participant in the nonclinical control group who did not complete the measure of trait paranoia.

We aimed to recruit as many clinical participants in the time available as possible. A sensitivity power analysis using G*Power 3.1^18^ revealed that our final sample size (*N* = 88; 46 men, 42 women) afforded 80% power to detect direct effects (H1-H4) with an effect size equal to or greater than *f*^2^ = .09 (equivalent to *r* = .29; a medium effect size). Tests of indirect effects (H5) tend to have more power than tests of direct effects^19^ and this power advantage of indirect effects increases for serial mediation models; “including multiple mediators may be more beneficial to power than including a single mediator or testing only the total effect for significance”.^20^

### Measures

#### Paranoia Scale^21^

This is a 20 item scale measuring trait paranoia. Participants rated the PS items (e.g., “someone has it in for me”) on a 5-point scale (1 = *not at all applicable to me*, 5 = *extremely applicable to me*), with higher scores indicating greater levels of trait paranoia (possible range 20–100). The measure is reliable (α = .84) and has been validated and used in both clinical and nonclinical samples.^21,22^ We summed the PS items to create a trait paranoia index.

#### State Paranoia Scale (SPS)^9^

This is a 4-item scale assessing state paranoia, which was developed specifically for use with the PDG. Participants rate how they perceive the other player by marking responses on a 7-point scale anchored with 2 opposing statements. The 4 paranoia items are: (1) “Is friendly towards me” vs. “Is hostile towards me”; (2) “Wants to please me” vs. “Wants to upset me”; (3) “Wants to help me” vs. “Wants to harm me”; and (4) “Respects me” vs. “Has it in for me.” All SPS items contain both elements of threat and intention, such that clear persecutory thinking was assessed. We summed the items to create an index, with high ratings indicating higher levels of state paranoia (possible range 4–28). The SPS has good internal consistency (α = .92).

#### Prisoner’s Dilemma Game (PDG)

**Fig. 2.**
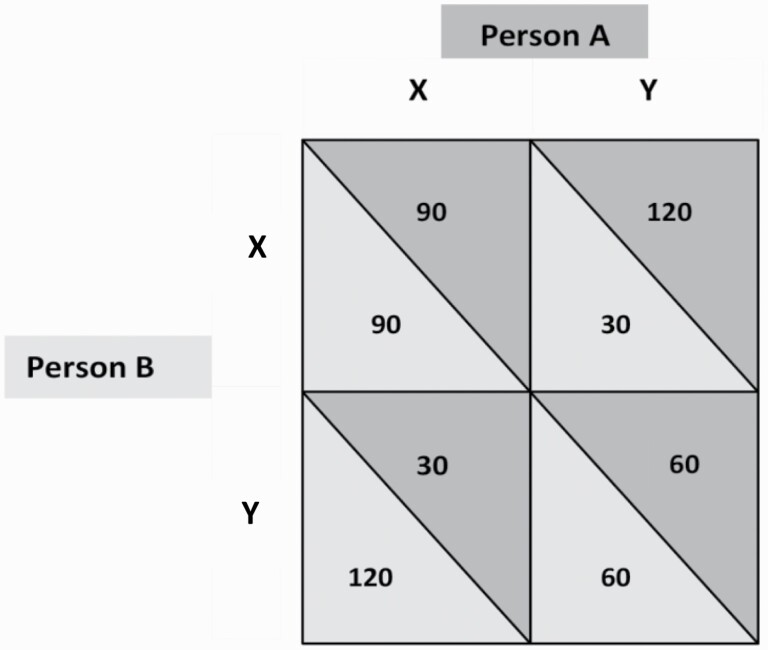
*The PDG Matrix. Note*. This figure shows the PDG matrix used in the study. X denotes cooperation and Y denotes competition. If both persons choose to cooperate (X), the payoff is 90 each; if both choose to compete (Y) the payoff is 60 each. If Person A competes (Y) and Person B cooperates (X), Person A gets 120 and Person B gets 30. If Person A cooperates (X) and Person B competes (Y), Person A gets 30 and Person B gets 120.

The PDG involves 2 players who make a simple forced choice either to cooperate with, or compete against, each other in the game. To play the game, participants are presented with a matrix (Figure 2) which summarizes the possible choices within the game (cooperate [labeled X in the matrix] or compete [labeled Y in the matrix]) and the associated payoffs they will receive. The payoffs received depend on whether each player chooses to cooperate or compete in the game. The dilemma is that each player can maximize their outcome by competing, yet paradoxically, when both players choose to compete, their outcomes are lower than the outcomes they can achieve by mutual co-operation. We indexed competition as the percentage of participants in each group (schizophrenia spectrum diagnosis vs. nonclinical controls) who chose to compete.

#### PDG Reasons Assessment^23^

This self-report measure comprises 10 items assessing various reasons for choice on the PDG. Each item is rated on a 7-point scale (1 = *not at all*, 7 = *very much*). For the purposes of this study, we were interested in the 2 items that measure distrust (“I wanted to defend myself against the actions of the other person” and “I did not trust the other person”) and the 2 items that measure greed (“I wanted to earn more than the other person” and “I wanted to maximize the difference between both persons in my favor”). We created composite measures of distrust and greed by summing the 2 relevant items (possible range 2-14; α =.70 and.71, respectively, for distrust and greed).

### Procedure

Participants first provided socio-demographic information, completed the Paranoia Scale, and then played the PDG. We modeled PDG procedures on prior research.^9,24^ We did not give participants specific information about their opponent or guidance on game strategy (e.g., to try and maximize ones’ earnings). In all information provided to participants, we labeled the two PDG choices simply as “X” and “Y” (it is only in writing about the research that we have adopted the terminology of “cooperate vs. compete”). We gave participants detailed instructions on the PDG matrix, including a review of the possible combinations of choices and their associated outcomes. Participants then had to pass a test of their understanding of the outcomes of various combinations of choices. We informed participants that they would be playing between 1 and 6 rounds of the PDG. We did so to ensure that they did not know that, in fact, there was to be only a single trial. When participants know that only a single trial is involved, this can increase competition (i.e., “end-gaming”), thereby producing restriction of range.^25^ After participants selected their PDG choice on the first (and only) trial, we administered the SPS and the reasons assessment. Finally, we debriefed participants and thanked them for their time.

## Results

### Sample Characteristics

We present descriptive statistics and zero-order correlations among study variables in table 1. Average age was significantly higher in the clinical group (*M* = 25.28, *SD* = 4.99) than in the control group (*M* = 21.11, *SD* = 3.39), *t*(86) = 4.53, *P* < .001, *d* = 0.97. Further, there were significantly more men in the clinical (*n* = 34) than control (*n* = 12) group, χ^2^(1, *N =* 88) = 18.09, *P* < .001, φ = 0.45. Therefore, we included age and gender as covariates in the mediation models.

**Table 1.** Descriptive Statistics and Correlations Among Study Variables

	*M*	*SD*	1	2	3	4	5	6	7
1. Schizophrenia	0.52	0.50	-						
2. Age	23.29	4.76	.44***	-					
3. Gender	0.52	0.50	.45***	.24^*^	-				
4. Trait paranoia	45.55	17.42	.50***	.21^*^	.23^*^	-			
5. State paranoia	15.10	3.59	.13	-.14	.00	.28**	-		
6. Distrust	7.32	3.63	.31**	-.08	.10	.50***	.43***	-	
7. Greed	8.66	3.91	.04	.04	.06	.07	.07	.30**	-
8. Competition	0.43	0.50	.24^*^	-.02	.01	.17	.17	.48***	.35**

*Note.* Schizophrenia was coded: 0 = control group, 1 = schizophrenia group. Gender was coded: 0 = women, 1 = men. Competition was coded: 0 = cooperate, 1 = compete. *N* = 88. Means for schizophrenia and gender reflect the proportion of participants in the schizophrenia group and proportion of male participants, respectively.

^*^ *P* < .05, ** *P* < .01, *** *P* < .001

### PDG Choice

Individuals in the clinical group competed significantly more in the PDG than those in the control group, χ^2^(1, *N =* 88) = 4.90, *P* = .027, φ = 0.24. In the clinical group, 21 participants cooperated and 25 competed on the PDG (54% competition); in the control group, 29 participants cooperated and 13 competed (31% competition).

### Mediational Analyses

All mediational analyses were conducted using the full sample of participants. First, we examined the individual direct effects specified in our theoretical model (H1-H4; table 2).

**Table 2.** Regression Analyses Testing Direct Effects Corresponding to H1-H4

	H1: Predicting trait paranoia			H2: Predicting state ­paranoia			H3: Predicting distrust			H4: Predicting competition		
Predictor	*b*(*SE*)	*t*	*b* ^*^	*b*(*SE*)	*t*	*b* ^*^	*b*(*SE*)	*t*	*b* ^*^	*b*(*SE*)	χ^2^	*b* ^*^
Schizophrenia	17.26 (3.98)	4.33***	.50	0.83 (0.98)	0.84	.12	1.34 (0.85)	1.58	.19	1.20 (0.71)	2.87	.33
Trait paranoia				0.06 (0.02)	2.49^*^	.29	0.08 (0.02)	3.58***	.37	-0.03 (0.02)	1.76	-.25
State paranoia							0.28 (0.09)	2.92**	.27	-0.02 (0.08)	0.07	-.04
Distrust										0.37 (0.10)	12.76***	.74

*Note.* Schizophrenia was coded: 0 = control group, 1 = schizophrenia group. *b* = unstandardized beta; *SE* = standard error; b* = standardized beta. The analysis testing H4 is a logistic regression analysis in which competitive choice was the dichotomous dependent variable (under H4: Predicting competition). We included gender and age as covariates in all analyses. *N* = 88.

^*^ *P* < .05, ** *P* < .01, *** *P* < .001.

**Fig. 3.**
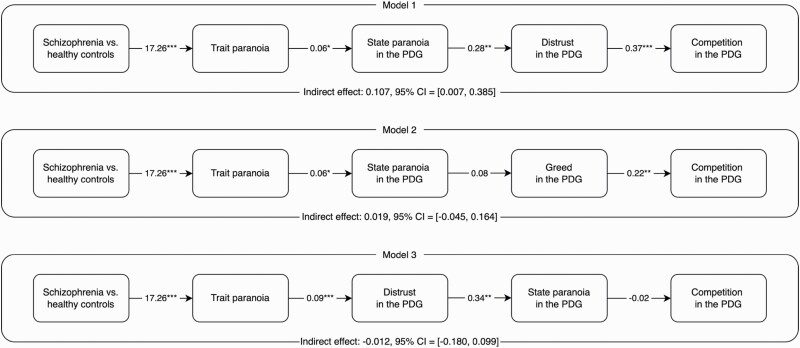
*The Hypothesized Serial Mediation Model (Model 1) and Two Alternative Serial Mediation Models (Models 2-3). Note.* Schizophrenia was coded: 0 = control group, 1 = schizophrenia group. Compared to Model 1, Model 2 substituted greed for distrust, and Model 3 reversed the order of state paranoia and distrust. Gender and age were included as covariates in all analyses. *N* = 88. * *P* < .05, ** *P* < .01, *** *P* < .001.

As hypothesized, schizophrenia (vs. control) was associated with higher trait paranoia (H1); trait paranoia predicted higher state paranoia in the specific context of the PDG (H2); state paranoia predicted greater distrust of the opponent in the PDG (H3); and distrust predicted more competition (H4). Therefore, each of the 4 individual hypothesized model paths was supported. Next, we used Hayes’ (2022) PROCESS macro (Model 6; 5,000 bootstrap samples)^26^ to test the serial indirect effect of schizophrenia on competition in the PDG via trait paranoia, state paranoia, and distrust (figure 3, Model 1). As hypothesized, the indirect effect was statistically significant, as the 95% CI did not include zero. Results supported the hypothesized serial mediation model (H5; figure 1).

### Testing Alternative Models

We tested alternative serial mediation models to examine the specificity of our theoretical model. First, we substituted distrust with greed in the model (figure 3, Model 2) to examine the pathway from schizophrenia to competition in the PDG via trait paranoia, state paranoia, and greed. We did not expect this model to be supported because, although greed was positively associated with competition in the PDG, schizophrenia (vs. control), trait paranoia, and state paranoia were not significantly correlated with greed (table 1). The 95% CI included zero and therefore the alternative serial indirect effect via greed was not supported. Second, we reversed the position of distrust and state paranoia, testing the pathway from schizophrenia to competition via trait paranoia, distrust, and state paranoia (figure 3, Model 3). We did not expect this model to receive support because state paranoia did not predict competition above and beyond distrust (see table 2, under H4). Indeed, the 95% CI for this serial indirect effect included zero.

## Discussion

Our study examined paranoia and competitiveness in individuals with schizophrenia experiencing current persecutory delusions and nonclinical controls. A significantly greater proportion of individuals with schizophrenia chose to compete in the PDG compared with nonclinical controls. We expected competition to be higher in the clinical group as it flows from the expectation that others hold malevolent intentions toward the self, a key defining characteristic of persecutory delusions. This demonstrates the importance of competition in the PDG for understanding paranoia in clinical (as well as nonclinical) populations. Our findings also add to the growing body of evidence demonstrating the utility of game theory paradigms for investigating paranoia^8–13^ and theoretical models that propose continuity between clinical and nonclinical experiences.^6,7^

Results supported our theoretical model. Direct effects for the individual paths in the model were significant, such that schizophrenia was associated with higher trait paranoia (H1), trait paranoia predicted state paranoia in the PDG (H2), state paranoia predicted distrust of the opponent in the PDG (H3), and distrust predicted competition (H4). Crucially, we found evidence for serial mediation, with an indirect effect of schizophrenia on competition in the PDG via trait paranoia, state paranoia, and distrust (H5). The 2 alternative models tested (substituting distrust with greed and reversing the position of distrust and state paranoia) were not supported. Collectively, this provides the first evidence for both a process model linking schizophrenia with competitiveness in interpersonal interactions (via trait paranoia, state paranoia, and distrust), and for the role of distrust in clinical paranoia. Future research could further scrutinize the role of distrust in paranoia across the continuum of experience by adopting other experimental paradigms that have been used to study paranoia or by using alternative methodology, such as naturally-occurring interpersonal transgressions.^8^

There are a number of limitations that warrant consideration. First, evidence for the serial mediation model was correlational and, hence, does not provide a strong basis for causal inferences. The limitations of the measurement-of-mediation design (i.e., designs in which the mediating variables are measured rather than manipulated) are well documented.^27^ Nonetheless, the design is informative because it puts the mediational hypothesis at risk. In the present case, the hypothesized serial indirect effect (H5) comprised four direct effects (corresponding to H1-H4). Failure to detect any one of these links would have resulted in rejection of the serial mediation model, yet each link held. Second, sample size was relatively modest, though this is typical in hard-to-recruit clinical populations, such as those with schizophrenia. A sensitivity power analysis indicated that the study was adequately powered to detect medium-sized effects. Third, the groups differed significantly in age and gender, though we controlled for these demographic variables in all hypothesis tests. Fourth, we did not collect information about psychiatric medications in the clinical sample and the study is therefore silent about whether medication was related to any of the outcomes measured. Finally, the sample consisted predominantly of individuals of white ethnicity and the findings may therefore have limited generalisability to individuals from other cultural backgrounds. Future research would do well to address this by recruiting larger and more representative samples.

Our findings have real-world relevance and significance. The PDG paradigm shows how people with persecutory delusions experience state paranoia and distrust in interpersonal interactions even with strangers who have no connection with their persecutory belief system. There are likely to be real-world negative consequences of this type of distrust and lack of co-operation for people with schizophrenia. Persecutory ideation appears to be particularly linked with loneliness^28^ and it has been argued that “paranoid symptoms imply a disruption of the processes involved in belonging and social trust”.^29^ The theoretical process modeled in the present study demonstrates in action how persecutory thinking can directly lead people to experience heightened state paranoia and ensuing distrust, resulting in a decision to compete rather than cooperate in social interactions with a stranger. Future research might explore in real world settings not only the impact such competitive behavior has on the person with persecutory ideation (e.g., social isolation and loneliness) but also on the other person in the dyad. For example, might it lead the interaction partner to withdraw and avoid the person with persecutory ideation, creating a vicious cycle that further exacerbates feelings of loneliness and lack of belonging?

In terms of clinical implications, the findings raise the important issue of how one can reduce persecutory ideation and its potential negative consequences, such as isolation and loneliness. One option is traditional cognitive behavior therapy for psychosis, although psychological reactance is easily triggered when working directly with persecutory ideation.^30^ A promising alternative is mindfulness for psychosis, which has been shown to benefit people with schizophrenia,^31^ including persecutory delusions,^32^ and does not require direct discussion of the content of persecutory beliefs. A novel third possibility is to strengthen personality traits that moderate paranoid ideation. For example, there is emerging evidence that trait forgiveness ameliorates paranoia in the nonclinical population.^8^ Future research could explore if interventions aimed at increasing interpersonal forgiveness disrupt the pathway from persecutory ideation to interpersonal competition observed in the present study.

To conclude, our study makes clear theoretical and methodological contributions. We provide the first evidence for a theoretical model by which schizophrenia leads serially to competitiveness in interpersonal interactions via trait paranoia, state paranoia, and distrust. Game theory paradigms, and the PDG in particular, are important for advancing theory and research in paranoia as it occurs across the continuum of experience.
